# Real world, multicentre patterns of treatment and survival in metastatic renal cell carcinoma with the UK Renal Oncology Collaborative (UK ROC): Is it time to look favourably on first‐line immunotherapy containing combinations in all IMDC groups?

**DOI:** 10.1002/cam4.7327

**Published:** 2024-06-21

**Authors:** John McGrane, Ricky Frazer, Amarnath Challapalli, Gihan Ratnayake, Zhaung Boh, Alison Clayton, Caroline Chau, Anand Sharma, Manal Elgendy, Natalie Charnley, Wael Mohamed, Sarah Kingdon, Andrew Protheroe, Anna Lydon, Anna Halstead, Vicky Ford, Iqtedar Muazzam, Dawn Lee, G. J. Melendez‐Torres, Amit Bahl

**Affiliations:** ^1^ Royal Cornwall Hospital Oncology Department Truro UK; ^2^ Velindre Cancer Centre Cardiff UK; ^3^ Bristol Haematology and Oncology Centre University Hospitals Bristol NHS Foundation Trust Bristol UK; ^4^ Musgrove Park Hospital, Somerset NHS Foundation Trust Taunton UK; ^5^ Edinburgh Cancer Centre, Western General Hospital Edinburgh UK; ^6^ The Northern Ireland Cancer Centre Belfast City Hospital Belfast UK; ^7^ University Hospital Southampton Southampton UK; ^8^ Mount Vernon Cancer Centre Northwood UK; ^9^ Newcastle Cancer Centre The Newcastle upon Tyne Hospitals NHS Foundation Trust Newcastle upon Tyne UK; ^10^ Royal Preston Hospital, Lancashire Teaching Hospitals Preston UK; ^11^ South Wales Cancer Centre Singleton Hospital Swansea UK; ^12^ Plymouth Oncology Centre University Hospital Plymouth NHS Trust Plymouth UK; ^13^ Oxford University Hospitals Oxford UK; ^14^ South Devon and Torbay NHS Foundation Trust Torquay UK; ^15^ University Hospitals Dorset Bournemouth UK; ^16^ Royal Devon University Healthcare NHS Foundation Trust Exeter UK; ^17^ Hull University Teaching Hospitals NHS Trust Hull UK; ^18^ University of Exeter Exeter UK

**Keywords:** favourable risk, immunotherapy, overall survival, renal cell carcinoma

## Abstract

**Introduction:**

Clinical trials show improved progression‐free survival (PFS) and overall survival (OS) in first‐line metastatic renal cell carcinoma (mRCC) patients with immunotherapy containing systemic anti‐cancer therapies (SACT). However, in the favourable international metastatic renal cell cancer database consortium (IMDC) group there is no trial evidence for OS benefit despite clear PFS improvement when comparing anti‐VEGF tyrosine kinase inhibitor (TKI) monotherapy and (immunotherapy and TKI) IO/TKI combinations.

**Objective:**

To assess the impact of first‐line SACT choice on the clinical outcomes of PFS and OS in mRCC. To evaluate this impact of initial SACT for allcomers and the favourable IMDC group.

**Methods:**

A multicentre retrospective review of patients who started SACT for mRCC (01/01/2018–30/06/2021) at 17 UK NHS trusts. Patient demographics and IMDC group were analysed. Survival data were compared using Kaplan–Meier curves, and the statistical significance of differences in outcome between the groups was assessed with the log‐rank test. Univariable and multivariable Cox proportional hazard modelling estimate the hazard ratios (HRs) for survival outcomes associated with IMDC and treatment subtype.

**Results:**

One thousand three hundred and nineteen patients were identified with a median age of 64. 294 (22.3%), 695 (52.7%) and 321 (24.3%) were IMDC group favourable, intermediate and poor, respectively. 311 (23.6%), 197 (14.9%) and 778 (59%) patients received checkpoint inhibitor and anti‐CTLA4 monoclonal antibody (IO/IO), IO/TKI and TKI first‐line SACT across all IMDC groups. Significant PFS improvement favouring IO/TKI versus TKI was demonstrated in allcomers HR = 0.61. In the favourable risk group, Log rank testing demonstrated a significant benefit for IO/TKI over TKI for PFS (HR = 0.60, 95% CI [0.39, 0.91]) and OS (HR = 0.42, 95% CI [0.18, 0.99]).

**Conclusion:**

In this real‐world evidence cohort, we have shown OS and PFS benefit with IO/TKI versus TKI in the favourable IMDC risk group. This has not been previously reported from trial outcomes and would support use of front‐line IO/TKI in mRCC favourable risk patients.

## Graphical abstract

Across all IMDC prognosis groups there was a benefit to combination therapy in the first‐line setting in terms of PFS when comparing IO/TKI versus TKI. Overall survival and progression‐free survival benefit was seen in the favourable IMDC risk group in this real‐world cohort for IO+TKI versus single agent TKI as first‐line SACT. Even in the IMDC favourable group 32.4% of patients did not make it to second‐line therapy.

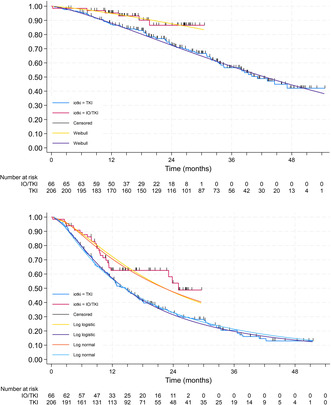

## 1 INTRODUCTION

There have been many major changes in the treatment landscape for metastatic renal cell carcinoma (mRCC) in the last 5 years. The two major changes have been the introduction of immunotherapy to the treatment paradigm and the increased use of combination therapies.

There have been multiple clinical trials showing benefit for combination immunotherapy containing therapies over traditional monotherapy anti‐VEGF tyrosine kinase inhibitor therapy (TKI) in the first‐line setting in mRCC. These combinations have all been compared as a combination therapy versus TKI monotherapy with sunitinib. These can be divided into immunotherapy with a checkpoint inhibitor and anti‐VEGF agent (IO/TKI) or a combination of immunotherapy with a checkpoint inhibitor and anti‐CTLA4 monoclonal antibody (IO/IO).^1^, ^2^, ^3^, ^4^, ^5^

The primary criteria for determining the treatment options for mRCC patients is the use of the international metastatic renal cancer consortium (IMDC) risk group.^6^ Although designed for prognostic reasons this has been used as a stratification factor for treatment choices in recent clinical trials. This has led to many international mRCC treatment guidelines being directed by IMDC risk group.^7^

Multiple trial data have demonstrated that there is a clear overall survival benefit in intermediate and poor IMDC risk groups for those first‐line patients receiving combination therapy versus single‐agent TKI. The same has not yet been shown in the favourable IMDC risk group where OS benefit has not been clearly demonstrated for combination therapy versus TKI therapy. This is thought due to the favourable IMDC group reflecting a biologically angiogenesis‐driven subtype that would benefit more from anti‐VEGF TKI therapy. This has led to equipoise among treating oncologists as to the optimal treatment approach for favourable risk patients. The intermediate and poor IMDC risk groups are thought to represent a more inflammatory tumour environment and a higher mutational burden and hence better response to immunotherapy.^8^, ^9^, ^10^

Chakiryan et al reviewed 5872 pts treated in the US looking at first‐line therapy and outcomes and showed that first‐line IO or IO/TKI combination had improved OS compared to TKI therapy patients. They also showed similar OS outcomes for IO/TKI and IO/IO treatment groups.^11^

Shah et al reviewed 1538 pts comparing axitinib and pembrolizumab versus ipilimumab and nivolumab or single‐agent TKI. This showed longer time on treatment with the IO/TKI combination 13.6 m vs 5.8 m versus 3.8 m, respectively.^12^

In a network meta‐analysis combining data from trials, survival benefit and efficacy was assessed between front‐line TKI monotherapy, IO/IO and IO/TKI combination therapies. No difference was found in OS between ipilimumab and nivolumab and axitinib and pembrolizumab in the full populations (HR, 1.34; 95% CI: 0.92–1.97). There was also no difference in PFS among the treatment groups.^13^, ^14^

In a subsequent network meta‐analysis, axitinib and pembrolizumab demonstrated a superior PFS and OS compared to ipilimumab and nivolumab in the full population, with no significant difference seen in the IMDC intermediate/poor risk population.^15^

The data published to date, supports the use of immunotherapy containing combinations as first‐line therapy for mRCC patients with clear OS benefit for intermediate and poor IMDC patients. There remains the question of OS benefit from immunotherapy in the favourable IMDC risk group either in up front combination in first line or indeed in any line of therapy versus VEGF TKI therapy.

### 1.1 Objectives

1. To determine the impact in terms of PFS and OS by initial treatment type—IO/IO, IO/TKI or TKI in allcomers.
2. To determine the survival outcomes in the favourable IMDC risk group by treatment type.

## 2 METHODS

A retrospective review of cases of mRCC were identified across 17 centres in the UK. The UK renal oncology collaborative (UK ROC) is a collaboration of UK NHS cancer centres collecting data for real world evidence in metastatic renal cancer patients.

Patients who started systemic anti‐cancer therapy for mRCC between 01/01/2018 and 30/06/2021 were included and patient characteristics such as gender, IMDC risk group, pattern of metastatic disease at presentation and lines of therapy were recorded.

This was a retrospective data collection study. Digital records were reviewed by a clinician and data anonymised to ensure that the study is conducted in accordance with the principles of all governance and GDPR regulations.

This study received ethics approval (REC reference 24/SC/0038) IRAS project ID 338935.

### 2.1 Eligibility criteria

#### 2.1.1 Inclusion criteria

Patients with a clinical, radiological or pathological diagnosis of metastatic renal cancer (mRCC) who commenced systemic anti‐cancer therapy (SACT) for mRCC between 01/01/2018 and 30/06/2021.

#### 2.1.2 Exclusion criteria

Patients under 18 years of age and mRCC patients who have started first‐line SACT outside of the timeframe of 01/01/2018–30/06/2021.

### 2.2 Statistical analysis

Survival data were analysed using Kaplan–Meier curves, and the statistical significance of differences in outcome between the groups were assessed using the log‐rank test.

Univariable and multivariable Cox proportional hazard modelling was used to estimate the hazard ratios (HRs) for survival outcomes associated with histologic and treatment subtype. Models were adjusted for IMDC risk group, age, SACT choice and patterns of metastases.

Progression‐free survival (PFS) was calculated from the date of starting first‐line SACT to the date of progression, and OS was calculated from the first‐line SACT to the date of death from any cause or, for surviving patients, to the date of last follow‐up.

To further characterise the impact of first‐line treatment types on disease outcomes, we used a single‐predictor Cox model to estimate hazard ratios (HRs). We also used a range of parametric distributions to describe the relationship between first‐line treatment type and disease outcomes and expressed these as time ratios (TRs).

Of the two disease outcomes, we then considered PFS more carefully due to its relative maturity as compared to OS. We used a causal inference method, inverse probability of treatment weighting, to better approximate the causal effect of first‐line treatment type on disease outcomes. We first constructed stabilised inverse probability weights using a vector of covariates known to be either prognostic for PFS or moderators of treatment effects, including sex, age at first treatment, clear‐cell histology, prior nephrectomy, brain metastases and bone metastases, in a logistic regression model for treatment assignment. We then re‐estimated the Cox proportional hazards model using a corrected sandwich variance estimation method.^16^

We also re‐estimated parametric survival regressions with the most appropriate distributions, defined by best visual fit and lowest Akaike information criterion (AIC) and Bayesian information criterion (BIC) using M‐estimation.^17^

Both variance estimation methods provide improved standard errors in the presence of uncertainly estimated weights, and overcome the limitations of robust standard errors, which are known to be unduly conservative in many scenarios.

This study was carried out along the STROBE guidelines for real world data reporting.^18^

## 3 RESULTS

One thousand three hundred and nineteen patients were included who met the eligibility criteria. Patients were predominately male (71%) with a median age at diagnosis of 64 years old (range 21–84). Patient demographics, tumour subtype and characteristics are summarised in Table 1. Median duration of follow up was 16 months.

**TABLE 1:** Patient and tumour characteristics.

Characteristics	*N* = 1319	IO/IO	IO/TKI	TKI	Misc/other
*n*	1319	311 (23.6%)	197 (14.9%)	778 (59%)	33 (2.5%)
Gender					
Male	937 (71%)	232 (74.6%)	142 (72.1%)	538 (69.2%)	25 (75.8%)
Female	382 (29%)	79 (25.4%)	55 (27.9%)	240 (30.8%)	8 (24.2%)
Age at diagnosis of metastatic disease	21–84 (median 64)	28–83	29–86	21–84	
IMDC group					
Favourable	294 (22.3%)	15 (4.8%)	66 (33.5%)	206 (26.5%)	
Intermediate	695 (52.7%)	200 (64.3%)	96 (49.0%)	380 (48.8%)	
Poor	321 (24.3%)	96 (30.9%)	34 (17.3%)	185 (23.8%)	
N/A	9 (0.7%)			7 (0.9%)	
First‐line SACT regimen		IpiNivo (100%)	AxiAve (85.3%)	Sun (41.6%)	
			AxiPem (14.2%)	Paz (30.1%)	
			LenPem (0.5%)	Cabo (14.7%)	
				Tivo (13.4%)	
				Axi (0.3%)	
Clinical trials involvement	44 (3.3%)	30 (9.6%)	0	5 (0.6%)	9 (27.2%)
Nephrectomy (excluding three cytoreductive = 718)	715 (54.2%)	144 (46.3%)	120 (60.9%)	434 (55.8%)	17 (51.5%)
Sarcomatoid changes	102 (7.7%)	48 (15.4%)	11 (5.6%)	43 (5.5%)	0
Clear cell component	1096 (83.1%)	247 (79.4%)	171 (86.8%)	650 (83.5%)	28 (84.8%)
Alive with median 16 month follow up	619 (46.9%)	165 (52.4%)	140 (70.0%)	296 (39.2%)	18 (54.5%)
Alive					
@12 m	68.9%	68.5%	77.1%	67.2%	
@18 m	60.2%	61.8%	69.4%	57.5%	
@24 m	52.3%	52.5%	62.5%	49.8%	

Abbreviations: Axi, Axitinib; AxiAve, Axitinib + Avelumab; AxiPem, Axitinib + Pembrolizumab; Cabo, Cabozantinib; IpiNivo, Ipilimumab + Nivolumab; Paz, Pazopanib; Sun, Sunitinib; Tivo, Tivozanib.

Eighty‐three per cent of patients had metastatic clear cell histology with papillary (5.6%) and unclassified (4.3%) the next two most common subtypes. 7.7% of patients had a sarcomatoid component in their histology.

Seven hundred and seventy‐eight patients had single‐agent TKI, 311 had IO/IO combination therapy and 197 had IO/TKI therapy as their first line of SACT. Thirty‐four patients had other investigational/ trial medication, so 1286 patients were analysed for this data.

### 3.1 To determine the impact in terms of PFS and OS by initial treatment type—IO/IO, IO/TKI or TKI in allcomers

#### 3.1.1 Allcomers—overall survival by first‐line treatment type

**FIGURE 1:**
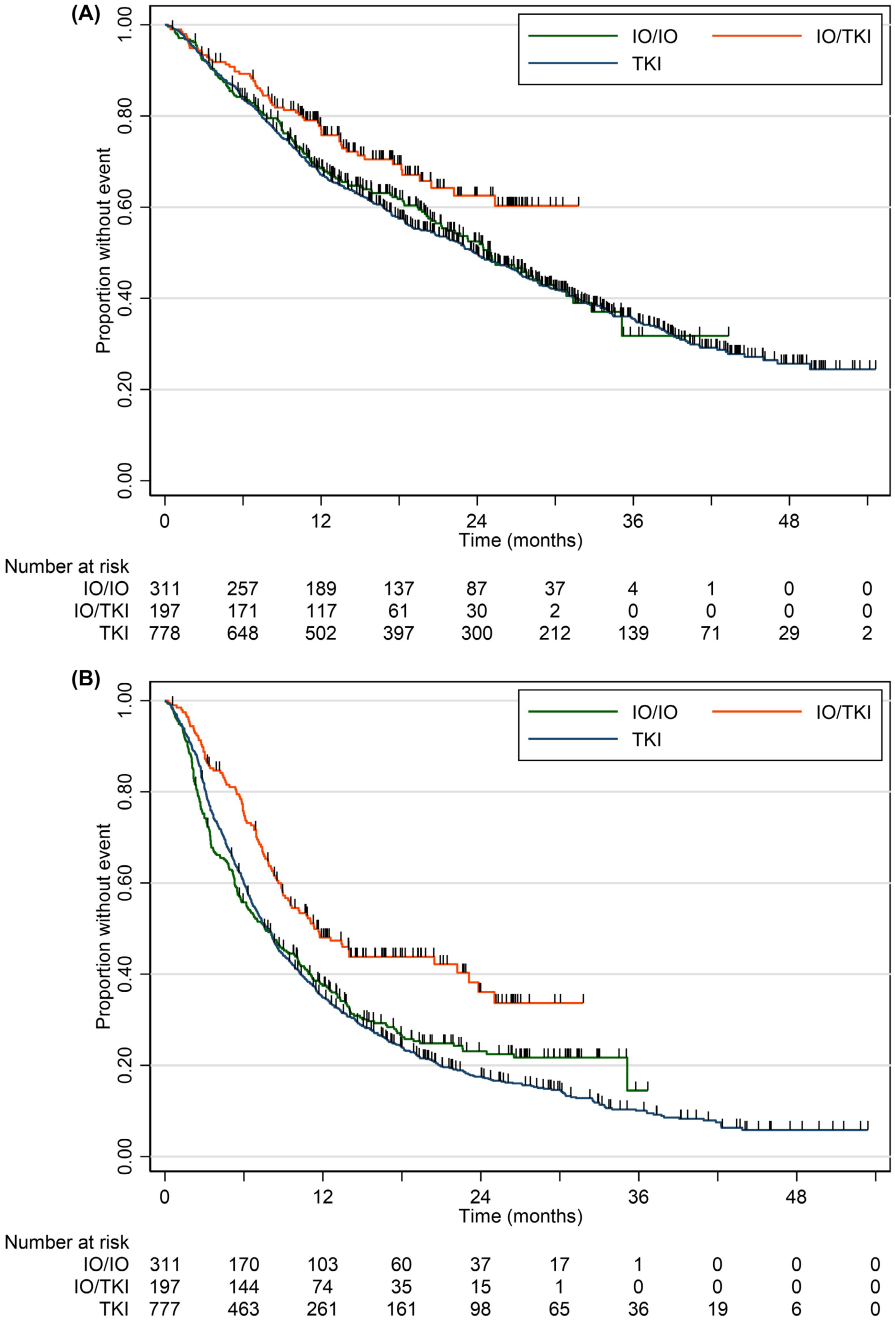
(A) Overall survival in Allcomers by first‐line SACT. (B) Progression‐free survival in Allcomers by first‐line SACT.

Median OS (months) for all patients receiving first‐line SACT was not reached in the IO/TKI group, 25.0 m in the IO/IO group and 23.8 m in the TKI group (95% CI [21.1, 26.9])—see Figure 1A.

In the IO/IO group 30.9% of patients were poor IMDC risk as opposed to 17.3% IO/TKI and 23.8% TKI.

Log‐rank testing for OS over the three categories suggested that the survival functions for OS were significantly different by group [χ^2^(2) = 7.69, *p* = 0.02]. Extending the results of the log‐rank test, the hazard ratio for IO/IO as compared to TKI was 0.96 (0.80, 1.16); for IO/TKI as compared to TKI, the HR was 0.68 (0.52, 0.90).

#### 3.1.2 Allcomers—progression‐free survival by first‐line treatment group

Median PFS for all patients receiving first‐line SACT was 11.3 m for IO/TKI (95%CI [9, 20.5 m]), 7.6 m for IO/IO (95%CI [6.2, 9.4 m]) and 7.8 m for TKI (95% CI [7.0, 8.4 m]).

A log‐rank test for PFS over the three categories suggested that the survival functions for PFS were significantly different by group [χ^2^(2) = 23.02, *p* < 0.0001]. Extending the results of the log‐rank test, the hazard ratio for IO/IO as compared to TKI was HR = 0.93, (95% CI 0.80, 1.08); for IO/TKI as compared to TKI, the HR =0.61 (CI 0.50, 0.75)—see Figure 1B.

### 3.2 To determine the survival outcomes in the favourable IMDC risk group by treatment type

#### 3.2.1 IMDC favourable group patients' overall survival by first‐line treatment type

Of the 294 favourable patients in the first line of SACT therapy 206 (70%) had TKI therapy, 66 (22.4%) had IO/TKI and 22 (7.4%) had other therapy.

In the IMDC favourable group patients the median OS was not reached in the IO/TKI group and 41.1 m in the TKI group (95% CI [34.4 m, not estimable]).

These analyses did not include patients receiving IO/IO combinations as these are not routinely provided for favourable risk patients in the NHS.

A log‐rank test for OS suggested a significant survival difference by treatment group [χ^2^(1) = 4.21, *p* = 0.04.] IO/TKI combinations delayed time to death as compared to TKIs (HR = 0.42, 95% CI [0.18, 0.99]).

To examine the presence of effect modification for IO/TKI and TKI, we used a multi‐predictor Cox proportional hazards model. We compared a model with terms for binary risk category and terms for treatment type against a term with interactions and compared these models using a likelihood ratio test. For OS, the model with an interaction was significantly better than a model without (*p* = 0.03), indicating greater effectiveness for the favourable risk group.

A test of Schoenfeld residuals (test of proportional hazards) yielded *p* = 0.88, and a log–log plot did not provide evidence of violation of proportional hazards which allows confidence in using the cox regression results (see Table 2).

**TABLE 2:** Estimates of IO/TKI versus TKI—overall survival.

Distribution	Time ratio (95% CI)	AIC	BIC
Exponential	2.86 (1.25, 6.55)	454.7	461.9
Weibull	1.96 (1.01, 3.78)	448.5	459.3
Log logistic	1.94 (1.05, 3.61)	450.9	461.7
Log normal	1.97 (1.06, 3.64)	462.9	473.8

**FIGURE 2:**
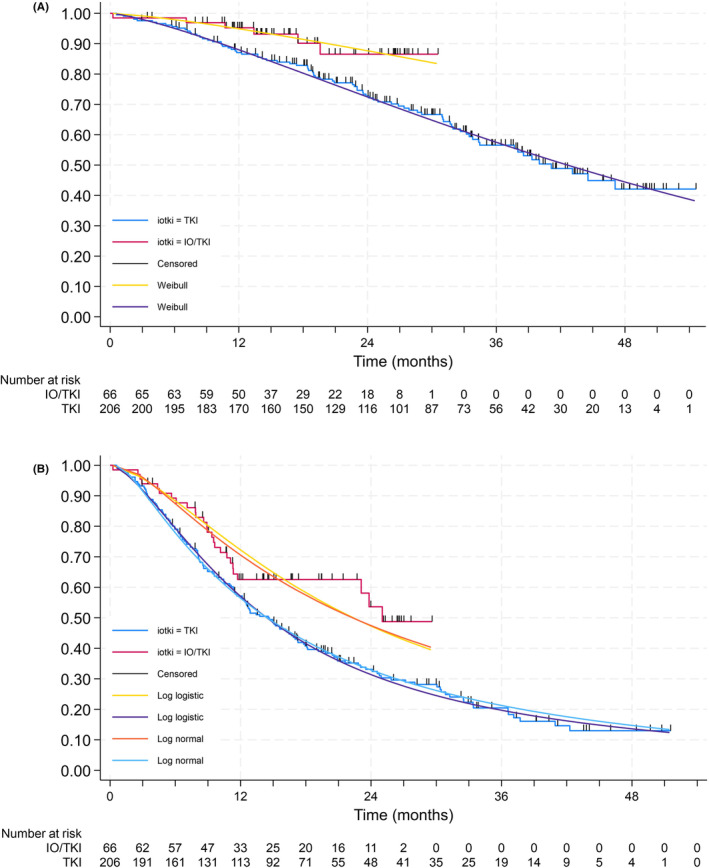
Overall survival (A) and progression‐free survival (B) for IO/TKI versus TKI in the IMDC favourable group (including Weibull distribution fit).

All parametric fits except for the exponential distribution generated consistent estimates of time ratio, each predicting that patients who received IO/TKI at first line lived nearly twice as long as patients who received TKIs. A Weibull distribution appeared to offer the best visual fit (see Figure 2) and had the lowest AIC and BIC across all distributions. This shows that patients on IO/TKI survived (OS) twice as long as those not on IO/TKI.

#### 3.2.2 IMDC favourable group patient's progression‐free survival by first‐line treatment type

In the IMDC favourable group patients' median PFS was 25.0 m in the IO/TKI group and 14.6 m for the TKI group (95% CI [34.4 m, not estimable]).

A log‐rank test for PFS suggested that PFS was significantly different by treatment type [χ^2^(1) = 5.85, *p* = 0.02]. IO/TKI combinations delayed time to death or progression as compared to TKIs (HR = 0.60, 95% CI [0.39, 0.91]).

To examine the presence of effect modification for IO/TKI and TKI, we used a multi‐predictor Cox proportional hazards model. We compared a model with terms for binary risk category and terms for treatment type against a term with interactions and compared these models using a likelihood ratio test, finding no significant evidence of interaction (p=0.57).

A test of Schoenfeld residuals yielded *p* = 0.93, and a log–log plot did not provide evidence of violation of proportional hazards. All parametric fits generated similar estimates of time ratio, each predicting that patients who received IO/TKI at first line spent at least 50% more time before progression or death (see Table 3). Both log logistic and log normal distributions offered the best visual fit (see Figure 2B) and had lower AIC and BIC values than exponential or Weibull.

**TABLE 3:** Estimates of IO/TKI versus TKI—progression‐free survival.

Distribution	Unadjusted			Adjusted
	TR (95% CI)	AIC	BIC	TR (95% CI)
Exponential	1.64 (1.08, 2.49)	705.5	712.7	
Weibull	1.50 (1.03, 2.17)	703.2	714.1	
Log logistic	1.55 (1.08, 2.22)	694.2	705.0	1.72 (1.16, 2.57)
Log normal	1.54 (1.07, 2.23)	696.8	707.6	1.61 (1.01, 2.56)

Checks of inverse probability of treatment weights did not reveal any extreme weights; all were within the interval [0.5, 2.2]. Re‐estimation of parametric survival regressions using estimated weights generated a more substantial effect of IO/TKI benefit versus TKI on PFS, suggesting an average increase of time spent before progression or death of between 61% and 72%. Similarly, re‐estimation of a Cox proportional hazards model generated a larger magnitude of benefit for IO/TKI (HR = 0.54, 95% CI [0.34, 0.86]).

#### 3.2.3 Patterns of treatment in the favourable IMDC group

The three most common TKIs used in the first‐line setting in the favourable setting were sunitinib (50.5%), pazopanib (31.6%) and tivozanib (15%). 95.5% of IO/TKI patients were treated with avelumab + axitinib (see Table 4).

**TABLE 4:** Lines of therapy based on first‐line therapy in the favourable IMDC group.

	Nivo	Cabo	Tivo	Len Eve	Axi	Paz	Sun	Other / trial	Axi Pem	Axi Ave	Len Pem
**1st line therapy TKI** *n* = 206		2.9%	15%			31.6%	50.5%				
2nd line therapy *n* = 113	57.5%	31%	4.4%	3.5%	1.8%	0.9%	0.9%				
3rd line *N* = 47	21.3%	61.7%		4.3%	12.8%						
4th line *n* = 13	7.7%	23.1%		7.7%	53.8%		7.7%				
**1st line** **IO/TKI** *n* = 66									3%	95.5%	1.5%
2nd line *n* = 23	0	52.2%	4.3%	30.4%				13%			
3rd line *n* = 6	0	33.3%		33.3%		16.6%		16.6%			

Abbreviations: Axi, Axitinib; AxiAve, Axitinib + Avelumab; AxiPem, Axitinib + Pembrolizumab; Cabo, Cabozantinib; LenEve, Lenvatinib + Everolimus; Paz, Pazopanib; Sun, Sunitinib; Tivo, Tivozanib.

Following IO/TKI, cabozantinib (52.2%) was the most common second‐line of therapy.

Censoring out 81 patients who were still on treatment 32.4% of favourable IMDC patients did not make it to second‐line SACT. If patients started on TKI therapy nivolumab (57.5%) was the most common second‐line therapy although 42.5% of patients were rechallenged with a further TKI agent with the majority of second‐line TKI being cabozantinib.

When looking at the 294 favourable risk patients, 97 (33%) died in the time frame of follow up. Fifty of the 97 patients who died (51.5%) had never received immunotherapy as part of SACT therapy.

#### 3.2.4 Survival by age category in the favourable IMDC group

We looked at the impact of patient age on PFS and OS in the favourable IMDC group by age category. There was a non‐significant trend towards improved OS favouring younger patients in both IO/TKI and TKI groups. PFS did not follow any clear pattern of difference by age group (see Appendix S1).

## 4 DISCUSSION

The landscape for mRCC continues to evolve. This offers patients real benefits in terms of cancer control and treatment options. In our large real‐world cohort, we have shown that the addition of immunotherapy to deliver combination therapy for first‐line mRCC treatment has significant overall and progression‐free survival across all IMDC prognosis groups.

In this manuscript we have not explored the survival differences between IO/TKI and IO/IO as most IO/IO patients are intermediate and poor risk groups which we know from historical datasets have worse outcomes. The fact that IO/IO group had 30.9% poor IMDC patients as opposed to 17.3% IO/TKI and 23.8% TKI would explain differences in OS for IO/TKI versus IO/IO or TKI in the allcomers cohort. The improved OS benefit for the favourable risk group is of particular interest given that trials exploring combination therapy versus TKI have not shown a significant OS benefit despite clear PFS benefits in clinical trials.^1^, ^2^, ^3^, ^4^, ^5^ We also tested the PFS benefit for cofounding variables such as sex, age at first treatment, clear‐cell histology, prior nephrectomy, brain metastases and bone metastases, in a logistic regression model for treatment assignment. This strengthened the case in favour of IO/TKI over TKI monotherapy. There were not enough patients with sarcomatoid features in the favourable risk group to explore that as a cofounding factor.

The OS benefit for IO/TKI versus TKI in the favourable IMDC group may reflect that in the real‐world setting patients are less fit with a higher disease burden than trial patients. The low rate of nephrectomy reflects this difference—54.2% compared to rates of 70%–86.3% in the clinical trials.^1^, ^2^, ^3^, ^4^, ^5^ Also, if trial patients are more robust, they may be more likely to receive subsequent lines of therapy to allow the opportunity for immunotherapy exposure in first‐line TKI monotherapy patients.

In our data, of the favourable IMDC patients who received first‐line TKI monotherapy, 42.5% did not receive immunotherapy in the second‐line therapy setting. This suggests there are a group of clinicians who support a TKI predominant approach in the favourable IMDC risk group. One explanation for this is a perception that multiple lines of therapy are possible in this risk group. The fact that 32.4% of IMDC favourable patients do not make it to second‐line therapy would challenge that view in our real‐world dataset.

The data presented suggests immunotherapy‐containing combination therapy improves OS as well as PFS in the favourable IMDC group. This supports combination therapy for all mRCC patients who are eligible to receive it. There will still be a cohort of patients where TKI monotherapy is appropriate due to comorbidity, contraindications to immunotherapy and patient preferences.

Ideally biomarker based treatment strategies rather than IMDC based would be the goal to optimise treatment selection and help optimise response from their first‐line therapy rather than IMDC group.^19^ The phase 2 BIONIKK trial has looked at a biomarker driven approach to first‐line SACT treatment selection which shows that biomarkers can be used to select patients who benefit from the addition of checkpoint inhibitor immunotherapy.^8^ The Meet‐URO 15 study has shown that adding further clinical and biochemical parameters to IMDC can allow for more accurate stratification of patients receiving nivolumab immunotherapy in the second line SACT.^20^

The predominate IO/TKI combination in this cohort was axitinib/avelumab as this was the only IO/TKI combination available that was UK NICE‐approved in the first line setting at the inclusion time period. The survival data for the JAVELIIN trial^3^ is the least mature of the IO/TKI trials. New IO/TKI combinations are available across mRCC practice but axitinib + avelumab remains the only IO/TKI combination available across England and Wales in the IMDC favourable risk group.

### 4.1 Strengths

Extensive modelling of confounding factors to give confidence in the robustness of representation of differences in survival in the favourable IMDC risk group was completed. This was done as this subgroup of patients have not been shown to have an OS benefit with addition of immunotherapy to upfront treatment regimens in phase 3 clinical trials. The PFS benefit was tested for cofounding variables such as sex, prior nephrectomy etc giving additional confidence in the benefit in PFS for IO/TKI over TKI.

This is a large cohort in the immunotherapy era with a large spread of NHS centres across the UK giving a good representation of the UK treatment landscape.

### 4.2 Weaknesses

This large UK data set has several limitations which are inherent to the real‐world aspect of data collection and analysis. This was a retrospective data collection. There was no data collected for response rate and limited data collected regarding treatment toxicity and patient comorbidity and its impact on treatment choice. We acknowledge that a prospective data collection including these parameters will add more information; however, as has been acknowledged by several real‐world data analysis across the spectrum of oncology these require robust infrastructure and support. Due to the evolving nature of this treatment space we now also have additional first‐line combinations which were not in routine use during the time frame of this study. Adjuvant pembrolizumab in the non‐metastatic setting^21^ is now available which may impact treatment choices if patients relapse.^21^

There may also be an element of selection bias whereby patients with comorbidities are being offered single agent TKI instead of combination therapy in the first‐line setting. We do not have a comorbidity index for patients challenge any selection bias.

Large scale prospective data collection in the real word setting hold promise to add rich insight into the first‐line SACT. The multi‐national European CARE‐1 study is looking at survival outcomes in mRCC by SACT choice in combination with routine biomarker, programmed death‐ligand 1 (PD‐L1) collection.^22^

Despite the limitations outlined, the UK ROC our data strongly supports the use of IO in combination across all IMDC subtypes. Where the only licenced options in the favourable risk patients are IO/TKI and TKI we strongly support the use of IO/TKI where possible. The OS benefit aligned with previously documented high rates of patient drop‐off between lines of therapy in mRCC (including the favourable risk groups) in similar cohorts which supports the use of the most effective treatment as first line SACT.^23^

### 4.3 Summary

1. Overall survival benefit was seen in the favourable IMDC risk group in this real‐world cohort for IO/TKI versus TKI as first‐line SACT.
2. Across all IMDC prognosis groups there was a benefit to combination therapy in the first‐line setting in terms of PFS when comparing IO/IO versus TKI and IO/TKI versus TKI.
3. A significant proportion of favourable risk patients did not receive immunotherapy at any point if they did not recieve it as part of their first‐line treatment.

## 5 CONCLUSION

Our real‐world data supports the choice of IO/TKI in the favourable risk group where possible and IO combination (IO/IO or IO/TKI) in the intermediate and poor IMDC risk groups as first‐line therapy for advanced RCC. Improvement in overall survival with IO/TKI in the favourable risk group could be practice changing.

### 5.1 Participating UKROC centres

1. Sunrise Oncology Centre, Royal Cornwall Hospital
2. Velindre Cancer Centre, Cardiff
3. Edinburgh Oncology Centre, Western General Hospital
4. Bristol Oncology Centre, Bristol Royal Infirmary
5. Musgrove Park Hospital, Taunton
6. Northern Ireland Cancer Centre, Belfast City Hospital
7. Mount Vernon Cancer Centre, Middlesex
8. Lancashire Teaching Hospital, Preston
9. University Hospital of Southampton
10. Newcastle Cancer Centre
11. Torbay & South Devon Foundation Hospital
12. Royal Devon & Exeter Hospital
13. University Hospital Plymouth
14. Oxford University Hospital
15. Hull Hospital
16. Pool Hospital
17. Southwest Wales Cancer Centre, Swansea

## AUTHOR CONTRIBUTIONS

**John McGrane:** Conceptualization (equal); data curation (equal); formal analysis (equal); investigation (equal); methodology (equal); project administration (equal); writing – original draft (equal); writing – review and editing (equal). **Ricky Frazer:** Conceptualization (equal); data curation (equal); formal analysis (equal); methodology (equal); writing – original draft (equal); writing – review and editing (equal). **Amarnath Challapalli:** Conceptualization (equal); data curation (equal); formal analysis (equal); writing – original draft (equal); writing – review and editing (equal). **Gihan Ratnayake:** Conceptualization (equal); data curation (equal); writing – review and editing (equal). **Zhaung Boh:** Data curation (equal); investigation (supporting); writing – review and editing (equal). **Alison Clayton:** Data curation (equal); investigation (supporting); writing – review and editing (supporting). **Caroline Chau:** Data curation (equal); investigation (supporting); writing – review and editing (equal). **Anand Sharma:** Data curation (equal); investigation (supporting); writing – review and editing (equal). **Manal Elgendy:** Data curation (equal); investigation (supporting); writing – review and editing (equal). **Natalie Charnley:** Data curation (equal); investigation (supporting); writing – review and editing (equal). **Wael Mohamed:** Data curation (equal); investigation (supporting); writing – review and editing (equal). **Sarah Kingdon:** Data curation (equal); investigation (supporting); writing – review and editing (equal). **Andrew Protheroe:** Data curation (equal); investigation (supporting); writing – review and editing (equal). **Anna Lydon:** Data curation (equal); investigation (supporting); writing – review and editing (equal). **Anna Halstead:** Data curation (equal); investigation (supporting); writing – review and editing (equal). **Vicky Ford:** Data curation (equal); investigation (supporting); writing – review and editing (equal). **Iqtedar Muazzam:** Data curation (equal); investigation (supporting); writing – review and editing (equal). **Dawn Lee:** Data curation (equal); formal analysis (equal); writing – review and editing (equal). **G. J. Melendez‐Torres:** Conceptualization (equal); data curation (equal); formal analysis (equal); methodology (equal); resources (equal); supervision (equal); writing – original draft (equal); writing – review and editing (equal). **Amit Bahl:** Conceptualization (equal); data curation (equal); methodology (equal); writing – original draft (equal); writing – review and editing (equal).

## CONFLICT OF INTEREST STATEMENT

All conflicts of interest for authors have been declared in the separate declaration of interests file attached. None of the authors have a conflict that has influenced their participation with this manuscript.

## ETHICS STATEMENT

This study has been reviewed by the NHS Health Research Authority under the research title: UK Renal Oncology Collaborative – Real word review of therapy choices and outcomes in metastatic renal cancer. REC reference 24/SC/0038. IRAS project ID 338935. NHS Health Research Authority. South Central – Hampshire B Research Ethics Committee 2 Redman Place Stratford London E20 1JQ. email: hampshireb.rec@hra.nhs.uk.

## PATIENT CONSENT STATEMENT

As this was anonymous retrospective data collected patient consent was not required. There was no human tissue or material handled as part of this project.

## PERMISSION TO REPRODUCE MATERIAL FROM OTHER SOURCES

All the figures, tables and data in this manuscript have been created by the authors involved for this manuscript. No external images have been used.

## Supporting information


Appendix S1.

## Data Availability

The corresponding author Dr John McGrane will be available to reply to queries and has no planned absence.
