# Supplementary material for: Real world, multicentre patterns of treatment and survival in metastatic renal cell carcinoma with the UK Renal Oncology Collaborative (UK ROC): Is it time to look favourably on first‐line immunotherapy containing combinations in all IMDC groups?
Source: Cancer Med. 2024 Jun 21;13(12):e7327. doi: 10.1002/cam4.7327 (PMC11192966; doi:10.1002/cam4.7327)
Supplement: Supplementary file 1 — Appendix S1. [file CAM4-13-e7327-s001.zip › cam47327-sup-0001-Supinfo.docx]

Appendix - IMDC Favourable group OS and PFS analysis by age group

Age category 0 = Age <60 years

Age category 1 = Age 60-75 years

Age category 2 = Age >75 years

Figure S1: Progression free survival in the IMDC favourable group by age category and treatment type

Figure S2: Overall survival in the IMDC favourable group by age category and treatment type
