# Supplementary figures and images for: Real world, multicentre patterns of treatment and survival in metastatic renal cell carcinoma with the UK Renal Oncology Collaborative (UK ROC): Is it time to look favourably on first‐line immunotherapy containing combinations in all IMDC groups?
Source: Cancer Med. 2024 Jun 21;13(12):e7327. doi: 10.1002/cam4.7327 (PMC11192966; doi:10.1002/cam4.7327)

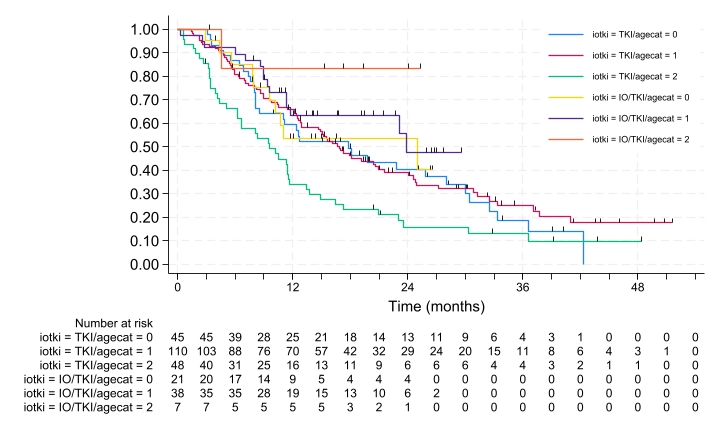

Supplement: Supplementary file 1 — Appendix S1. [file CAM4-13-e7327-s001.zip › cam47327-sup-0002-Appendixfigure1.jpg]

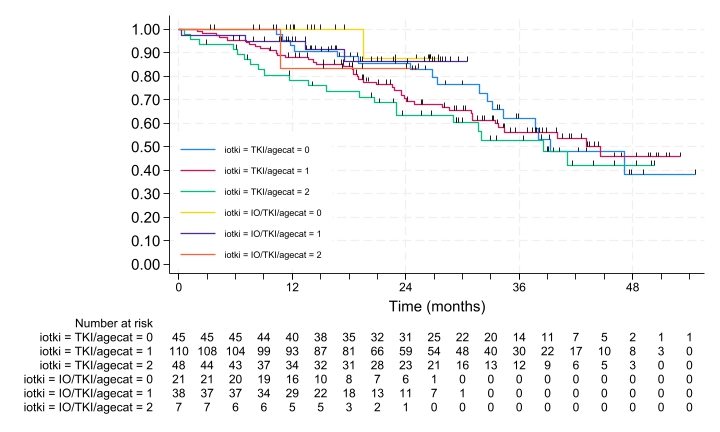

Supplement: Supplementary file 1 — Appendix S1. [file CAM4-13-e7327-s001.zip › cam47327-sup-0003-Appendixfigure2.jpg]
